# Mitigating the Impact on Users’ Privacy Caused by over Specifications in the Design of IoT Applications

**DOI:** 10.3390/s19194318

**Published:** 2019-10-06

**Authors:** Alfredo Pérez Fernández, Guttorm Sindre

**Affiliations:** Department of Computer Science, Norwegian University of Science and Technology (NTNU), NO-7491 Trondheim, Norway; guttorm.sindre@ntnu.no

**Keywords:** privacy, privacy-by-design, IoT, ubiquitous computing, internet of things, HCI, goal-oriented analysis, overspecification

## Abstract

Privacy has long been an important issue for IT systems that handle personal information, and is further aggravated as technology for collecting and analyzing massive amounts of data is becoming increasingly effective. There are methods to help practitioners analyze the privacy implications of a system during the design time. However, this is still a difficult task, especially when dealing with Internet of Things scenarios. The problem of privacy can become even more unmanageable with the introduction of overspecifications during the system development life cycle. In this paper, we carried out a controlled experiment with students performing an analysis of privacy implications using two different methods. One method aims at reducing the impact of overspecifications through the application of a goal-oriented analysis. The other method does not involve a goal-oriented analysis and is used as a control. Our initial findings show that conducting a goal-oriented analysis early during design time can have a positive impact over the privacy friendliness of the resulting system.

## 1. Introduction

Privacy is important to address in any system handling personal data, but this is often quite challenging. The problem starts from the very definition of privacy, many authors are often cited with a one line authoritative sentence explaining what privacy is [1,2,3], but, in words of Solove, “privacy is a plurality of different things and that the quest for a singular essence of privacy leads to a dead end” [4] (p. ix). Despite the difficulties of providing a satisfactory definition of privacy, it has been regarded as a concerning issue for the end users, even though they might behave as if it was not [5]. This concern is escalated by technologies that can collect personal data much more massively than before, such as Internet of Things applications [6], especially if the technologies are new and unknown to the users [7]. Legal developments like the General Data Protection Regulation (GDPR) also indicate that privacy is of growing public and political concern. We consider that privacy threats occur when personal information is disclosed in excess. This information disclosure is either purposeful (when there is at least one individual actively taking actions so that the disclosure happens) or incidental (when none of the actors involved in the event had the intention of actively disclosing the information). Examples of purposeful privacy threats can be those in which a corporation collects personal data for processing purposes of any type, whereas incidental disclosures are mainly caused by a faulty design of a system. The mechanisms to protect users’ privacy are different depending on whether they are aimed at preventing purposeful threats or incidental threats. On the one hand, the way to prevent purposeful threats is by establishing dissuasive regulations enforced by law and making sure that these regulations are known, understood, and obeyed. On the other hand, the way to prevent incidental threats caused by faults in the design of a system is by implementing privacy aware systems. According to Hoepman [8], there are two main classes of strategies to accomplish this, and they are either data-oriented, such as privacy-by-architecture [9,10,11,12], or process-oriented, such as privacy-by-design [13,14,15,16]. Our focus is mainly on process-oriented strategies, which consist on using methods, processes or frameworks that guide the design process of the system itself. It is known that overspecifications (also referred to as over requirements), defined as the “tendency to specify, design and develop a software system beyond the actual needs of the customer or the market” [17,18], tend to occur at any phase of the project life cycle, and the negative impact that they have includes launch delays, increased allocated resources (time and budget), and reduction of the software quality (high complexity, low reliability and poor maintainability). As indicated by Shmueli et al. [18], this phenomenon has been poorly covered by the literature, and there is no evidence that it has been studied with respect to how the resulting design might have a negative impact on maintaining the privacy of the users of Internet of Things (IoT) applications.

As overspecification is a trap that system developers often fall into, it is important to provide them with methods and guidelines that can help them avoid it. In previous papers we reported on the Privacy Aware Transmission Highway (PATH) framework [19], which is supposed to help developers identify privacy challenges in a system-to-be to avoid overspecification by making clear what privacy related design decisions are really mandated by the users’ privacy requirements. The PATH framework is an iterative process framework consisting of four phases: goal-oriented analysis, elaboration of the privacy related, evaluation or estimation of privacy impact, and iteration. The initial phase (goal-oriented analysis) is specifically designed to address overspecifications. The other phases are focused on privacy impact estimation and selection of design alternatives. An overview of PATH framework is presented in Section 3.2, whereas a much more detailed description is available in our previous publication [19] The guidelines of the PATH framework have been validated with experts in ubiquitous computing systems and HCI practitioners [20]. Unfortunately, most software development projects do not have privacy experts in their teams. Rather, design specifications—also of systems where privacy is a key concern—tend to be made by generic software developers with limited knowledge about privacy [9,21]. Therefore, an important test for the PATH framework is whether it can be understood and used effectively by software developers lacking any expertise in privacy. The idea of this paper is thus to carry out a controlled experiment where participants use PATH to solve a design-related task, and study the outcomes to see if they were able to use PATH effectively. More specifically, we want to investigate the following research questions:

RQ1: Can PATH be used effectively by people knowledgeable in general software development methods, but without expertise in privacy or privacy-enhancing technologies, to reduce the kind of overspecifications that might hurt user privacy?

RQ2: What type of design decisions with potential negative impact on privacy should a privacy framework such as PATH help identify? 

Considering the pros and cons of the various research methods, an experiment was found to be the most viable approach at the current stage. Compared to the questionnaire survey, the experiment gives data about actual usage of the framework, rather than just beliefs about its usability. Compared to the case study, the increased researcher control of the experiment means that it is safer and can give quicker results, and findings from the experiment might later be used as preparation for a more ambitious case study. In Section 2, we present the related work. Section 3 describes the research method that has been applied. The results of the controlled experiment are presented in Section 4. Section 5 discusses the findings. Possible threats to validity are considered in Section 6. Finally, Section 7 presents concluding remarks and elaborates on future work.

## 2. Related Work

Boehm [22] used the term “gold plating” as a metaphor to refer to the situation in which a project manager or developer incorporates features to the project beyond those being requested by the customer. Boehm proposes the win–win spiral model, based on the Theory W (win–win), to address this issue by encouraging the identification of stakeholders and their win conditions on each iteration of the spiral during the development process. Jones [23] refers to the changing requirements phenomenon as requirements creep, caused by the lack of understanding of the problem domain by the system developers. Jones proposed to reduce the impact of changing requirements by up to 25–50% by applying techniques such as Joint Application Design (JAD), early prototyping, or Rapid Application Development (RAD). Yu and Mylopoulos recommended applying a goal-oriented requirements engineering technique, by repeatedly asking *why*, *how*, and *how else* questions, as a solution to make it “[...] less likely to overspecify by prematurely committing to certain technological solutions” [24]. Buschmann [25] identified three common problems in the software engineering industry: featuritis (the sacrifice of quality in favor of excessive functionality), flexibilitis (the sacrifice of quality in favor of excessive flexibility), and performitis (the sacrifice of quality in favor of excessive performance). Buschmann prescribes the usage of a baseline architecture (named walking skeleton) with the minimal functionality needed to target the success scenario so that the cost of architectural changes can be reduced. Coman and Ronen [26] consider that overspecifications and over design are highly negative phenomena in the software industry and identify the main reasons why they appear. They recommend following some prescriptive approaches such as applying Simon’s satisficer [27] approach (focusing on the 20% of the features that account for 80% of the profits). Shmueli et al. [28] determined through an empirical study that the outside-view approach helps prevent the appearance of overspecifications. Other authors analyze more in detail the problem through case studies [29,30,31] or empirical studies [32,33].

There are also frameworks that address the problem of privacy more specifically, even though they do not cover the topic of overspecification. One of the most broadly known is the FIPS [34], which is commonly used in the industry mainly because of its simplicity and generality. Bellotti and Sellen [35] adapted MacLean et al.’s framework [36] Questions–Options–Criteria (QOC) to their Feedback/Control(FC) process in order to help identify personal privacy flaws in audiovisual media spaces. This framework (QOC-FC) became highly influential and served as a basis for further research in the design of privacy frameworks in ubiquitous computing. Jensen et al. [37] emphasize the importance of conducting privacy analysis in an iterative manner. The privacy requirements distillation process [38] is intended to be used to obtain a list of privacy related requirements when developing a mobile app. ElShekeil and Laoyookhong [39] implemented APSIDAL, a process framework used to operationalise the requirements of privacy-by-design imposed by the GDPR.

As it has been discussed, we found two different types of related work relevant to our research. On the one hand, there has been some research on overspecification and methods to avoid it, but these have not focused on privacy. On the other hand, there are some methods/guidelines related to design for privacy and in the area of IoT and ubiquitous computing, but these have not focused on the mitigation of overspecification. Our contribution—the PATH framework—aims at establishing a bond between both research areas, with the objective of mitigating the impact on users’ privacy caused by overspecifications in the design of ubiquitous computing systems.

## 3. Research Method

According to the research questions, our intention is to find out whether PATH can be effectively used by practitioners knowledgeable about software development, yet lacking expertise in privacy, and what design issues related to privacy the framework helps them identify. This points towards an empirical research method, for which there would be several different options:

- Questionnaire survey [40]: A description of PATH could be given to persons in the target category (i.e., knowledgeable about software development, yet not being privacy experts), together with some questions exploring whether they find the guidelines of PATH understandable and whether they think it will help them avoid overspecification. The advantage of a questionnaire survey is that one might reach a high number of respondents quickly, and thus get a big amount of data. The main weakness is that findings from questionnaires might be shallow and would mainly be about the respondents’ perceptions of the understandability and effectiveness of PATH. For example, a respondent who reads the guidelines might think they are understandable and respond positively about its believed effectiveness, whereas the actual usage of the framework might have uncovered that there were guidelines the participant was unable to follow or important issues misunderstood. A particular weakness about questionnaires for our RQs is that questions would be somewhat hypothetical, making it hard for respondents to answer reliably (e.g., it is hard to estimate the effectiveness of a method you have not tried). Therefore, the questionnaire approach was not chosen for this research, as the disadvantages and shortcomings were considered to far outweigh the possibility to get more data quickly.
- Case study [41]: For example, having somebody use PATH in a software development project for a considerable period of time, with several approaches to data collection along the way (e.g., observing them at work, interviewing them, studying design documents, and developed software). This would indeed have been very interesting, especially if it were a real industrial software project where privacy issues was a key concern. However, it is difficult to convince someone to use a yet untried framework in a real project. Also, such a case study would be quite time-consuming. Therefore, such a type of evaluation was considered overambitious at the current point. It would be more feasible to get some low threshold evidence for the effectiveness of the framework first, and then possibly build on that for more ambitious evaluations.
- Experiment [42], with recruited participants, performing a rather short duration task using the PATH framework, as administered by the researcher. The advantage of such an experiment compared to the case study is that it is easier to conduct because of the short duration, and the researcher has more control, e.g., about what task the participants shall perform. It is also regarded as the classical method to establish a cause–effect relationship [42]. However, the increased control of the experiment, comes at the cost of reduced realism, as participants can only be kept in a controlled setting for a limited period of time; this means that the task must be fairly small, and thus more limited in scope than a typical industrial software development task.

At the current point of the research, and with the given research question, an experiment was found to be the most reasonable approach compared to the other alternatives. The next question is then to decide the type of experiment. The possibilities would be

1. Having all participants use PATH only.
2. Comparing PATH (as the new treatment) to some kind of control. This control might be:
  (a) using just an ad hoc common sense approach, i.e., no method at all related to privacy issues, and
  (b) using another privacy method, preferably one that was well established or the most suitable one could find.

Option (1) would have the advantage of producing more usage data about PATH with a given time-frame and number of participants, as a percentage of the students would not be using other approaches as a control. However, a disadvantage is that it would then only produce absolute data, which would be hard to compare to anything. It could have worked adequately if there existed well-established benchmarks or metrics for productivity on the tasks in question, but this is not the case—as established in the Related Work section, there are few previous methods combining privacy with the mitigation of overspecification. Therefore, running an experiment with PATH only and finding that participants discovered X privacy issues within an hour of usage, it would be hard to know whether that was a good number, thus hard to draw any conclusions from the experiment. For this reason, the usage of a control appeared like a better idea. The ad hoc alternative was discarded for fear that it would too easily have PATH come out positively. Unless a method is poorly specified or unfit for the task, one would expect that the usage of a dedicated method would help performing a task better than the ad hoc approach, especially when participants are not experts. Therefore, comparing PATH with another method was chosen as the most viable approach. The method chosen as control was QOC-FC from Bellotti and Sellen [35] due to its simplicity [14], and because it has been used as a comparison benchmark in similar experiments before [37].

For this experiment, we decided to recruit final year Bachelor students. Many might argue that experiments trying out new design techniques would be more credible if using practitioners than students [43]. However, several studies have indicated that students can be good proxies for practitioners, e.g., the authors of [44,45], and Sjøberg et al. [42] specifically state that experiments with students are a appropriate way to test initial hypotheses. The Bachelor students participating in our experiment were just some months away from finishing their degrees, whereupon some would go into the workforce and some to master studies. Therefore, their competence would not be very different from that of novice practitioners with Bachelor degrees. Particularly relevant for our study, Salman et al. [46] found that for development approaches that were new both to students and professionals, the two groups performed similarly. The fact that the approach would be new was key to our research questions, as we wanted to check whether people with limited experience in privacy would be able to use the PATH framework. Having participants that were all students at the same stage in the same study program, and exposed to the same package of courses, made it easier to control this than what would have been the case with practitioners, where backgrounds might have been more diverse according to projects they had been involved in at work. Nevertheless, as Sjøberg points out, “[t]here are good reasons for conducting experiments with students as subjects, for example, for testing experimental design and initial hypotheses[...]” [42], which is our case.

With respect to how the students should work with the experiment task, there were two possibilities: having the students complete the task individually or in groups.

- An individual set-up has a number of advantages. The number of data points would be higher, making it easier to find statistically significant results. Also, some research indicates that, during brainstorming, individuals might be able to achieve a higher productivity, in terms of quantity and quality of the generated ideas, than if they are arranged in groups [47,48].
- A group-based set-up seems more realistic in industrial software projects. This type of design work and decision-making process is typically done by a team larger than one individual [49].

Iachello et al. described the benefits of second-generation methods; those that promote collaboration between system designers and the exchange of points of view, instead of simply following repeatable and mechanical procedures (which are first generation methods) [50]. As the privacy frameworks themselves should promote collaboration among the designers, we considered that a collaborative context was a more appropriate way to conduct the experiment. For this reason, we decided to choose a group-based setup with three or four individuals per group.

In our experiment, 14 last year Bachelor students in Informatics were recruited on a voluntary basis in exchange of an economical compensation. The payment was not made to each student personally, but to a nonprofit student social organization of the participants’ choice. Eleven students were male and 3 female within an age range from 20 to 26 years. A more even gender distribution would have been desirable, however, the obtained distribution was representative of the study program and also approximate to the distribution in the professional sector.

The experiment took place in two different sessions on consecutive days: Thursday and Friday from 10:00 to 14:00. Those attending the first day were explicitly requested to avoid sharing information about the experiment with anyone participating the day after. Each session started with a brief description of the task to address (with a duration of 30 min), followed by an introduction of the method to be used by the students (with a duration of 30 min). After the introduction, the students were split in two groups of approximately the same size: A and B on the first session and C and D on the second session (Table 1). The groups were requested to perform the same task, based on the description of the problem (Section 3.1) and elaborate a list of design alternatives after following the proposed methods to address privacy issues: QOC-FC [35] and PATH [19]. A and B were requested to use the QOC-FC method and C and D the PATH method, so that intergroup consistency could be validated. The most significant difference between both frameworks is that PATH includes a preliminary phase of goal-oriented analysis (GOA) based on the proposal of [24]. Both frameworks are described in detail in Section 3.2. The analysis performed is described in detail in Section 3.3.

### 3.1. Assignment Description

**Figure 1:**
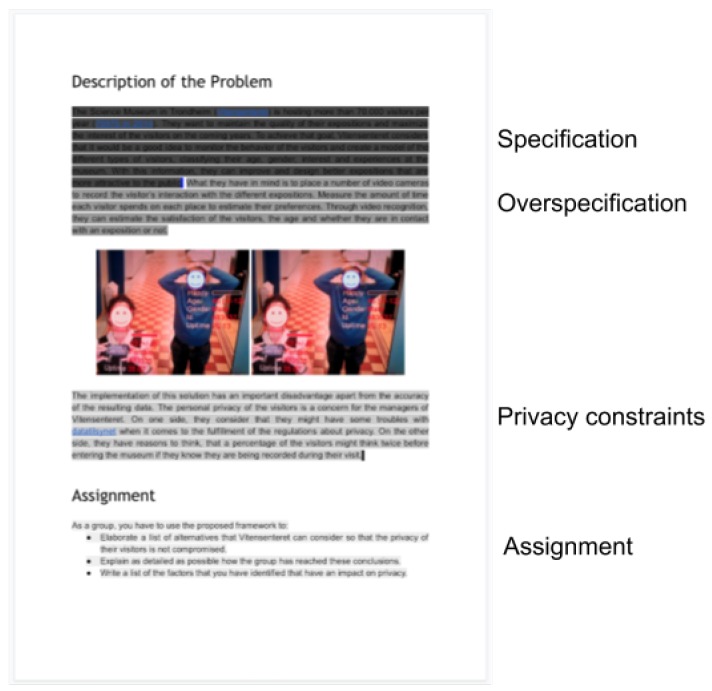
Structure of the problem description.

The students were given a one-page description of the problem to analyze (Figure 1). The description was based on an actual need presented by The Science Museum in Trondheim (Vitensenteret), namely, to collect information from their visitors to improve the quality of their exhibitions accordingly and align the design to the needs of the users. The problem description deliberately introduced an overspecification in the fifth sentence of the first paragraph, by stating that the initial consideration of the museum was to use video recording and computer vision techniques in order to measure the engagement and satisfaction of the visitors. An image was included in the description where two visitors appear in front of the camera. A label was displayed by the system indicating the estimation of five attributes corresponding to each visitor: happiness, age, gender, customer id, and presence time.

The second paragraph introduced the requirement of taking into consideration the users’ privacy. This requirement was presented from two different perspectives: first, the regulatory perspective of privacy (by stating that the organizers needed to comply with the applicable privacy related regulations) and, second, the aspect of privacy as a concern of the users that could feel bothered by being recorded. The assignment for the students was to use one of the frameworks mentioned in Section 3.2, to analyze the scenario and elaborate a list of alternatives for the Science Museum to consider in their final design. The problem description document had 301 words in total, distributed over the four sections: specification (95 words), overspecification (61 words), privacy constrain (87 words), and assignment (58 words).

### 3.2. Privacy Frameworks

**Figure 2:**
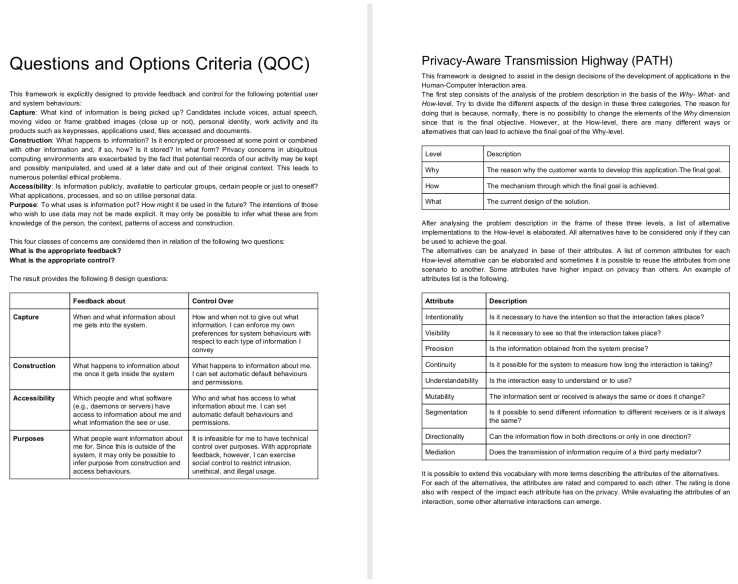
Summary of the QOC-FC framework (**left**) and the PATH framework (**right**).

The two frameworks, QOC-FC and PATH, were summarized in a one-page document each. Groups A and B were given a paper copy of the QOC-FC framework and groups C and D were given a copy of PATH. Both documents had a similar length in terms of words QOC-FC was 392 words (Figure 2, left) long and PATH was 402 (Figure 2, right).

The QOC-FC framework presented four main concepts based on users’ concerns about privacy related to the behavior of the system, Capture, Construction, Accessibility and Purpose:

- Capture: What kind of information is being picked up? Candidates include voices, actual speech, moving video or frame grabbed images (close-up or not), personal identity, work activity and its products such as key presses, applications used, files accessed, and documents.
- Construction: What happens to the information? Is it encrypted or processed at some point or combined with other information and, if so, how? Is it stored? In what form?
- Accessibility: Is information publicly, available to particular groups, certain people or just to oneself?
- Purpose: What is the information used for? How might it be used in the future? Is it going to be combined with information from third parties?

Two questions are formulated for each of these concerns: What is the appropriate feedback? What is the appropriate control?

The description of the PATH framework consisted of two parts. The first part was 134 words long and it explained how to conduct a simplified Goal-Oriented Analysis (GOA) by separating the design decisions into three different levels, the why (The reason why the customer wants to develop this application, the goal), the how (The mechanism through which the goal is achieved) and the what (The actual design). The second part of the framework was 268 words long and it proposed to analyze each of the how-else-level alternatives (interaction mechanisms), identified depending of their estimated attributes and the impact they might have on the users’ privacy, Intentionality, Visibility, Precision, Continuity, Understandability, Mutability, Segmentation, Directionality, and Mediation.

- Intentionality: Is user intent necessary for the interaction to take place?
- Visibility: Is it necessary to see that the interaction takes place?
- Precision: Is the information obtained from the system precise?
- Continuity: Is it possible for the system to measure how long the interaction is taking?
- Understandability: Is the interaction easy to understand or to use?
- Mutability: Is the information sent or received always the same or does it change?
- Segmentation: Is it possible to send different information to different receivers or is it always the same?
- Directionality: Can the information flow in both directions or only in one direction?
- Mediation: Does the transmission of information require of a third party mediator?

The students that used QOC-FC (Groups A and B) participated in the experiment on a different session than the students that used PATH (Groups C and D). This was arranged like this to avoid a possible diffusion of treatment (i.e., participants in the treatment group affected by participants in the control group, or vice versa). We considered that the risk of diffusion of treatment was higher among groups within the same session (synchronously) than among groups from different sessions (asynchronously, one group on Wednesday leaking details of the experiment to another group before the session on Thursday). For us, it was more important to guarantee the isolation of PATH and QOC-FC groups than to achieve a more robust intergroup consistency. Ideally, the experiment could have been conducted in four isolated rooms with one group per room. We did not consider this as an option, as that would have made it more difficult for us to maintain control of all the rooms at the same time (i.e., answering questions from students in an appropriate way, making sure they do not get distracted from the task or observing specific aspects of the collaboration).

### 3.3. Data Analysis

The purpose of the data analysis is two-fold. First, to find differences in the patterns of interaction mechanisms mentioned by the students in the interview, depending on the privacy framework used for the evaluation. Second, to identify potential new categories of privacy related concepts that are not considered beforehand. The reasoning for this analysis is that the dependent variable, the number of mentions to the overspecified requirement of using video capture based interaction mechanisms, is expected to be higher for those participants that are not requested to perform a GOA of the problem description, which is the independent variable. The approach adopted to analyze the data is the Content Analysis as described in [51]. The interview is first transcribed into text and then labeled applying the following coding schema based on the keywords video capture (video recording, cameras, camera, or recording), feedback Terminal (happy-or-not button, security check airport terminal, or happy buttons), ticket-based (ticket, entrance, or card), presence sensors (Touch sensors or infrared reflective sensors), BCI, Wifi, Bluetooth, RFID, NFC, and QR-Code.

We chose not to limit the process of coding to counting only the exact keywords. Instead, the focus of the coding was on the subject (in this case the interaction mechanism). This requires a more interpretative approach, to consider not only manifest content but latent content as well [51] (p. 297). Even though this can be seen as a threat to maintaining a proper inter- and intracoder reliability; in our case of study, there should be little to no disagreement with respect to which interaction mechanism the students are referring to. Another decision that was adopted was to count the number of interventions in which an interaction mechanism was referenced one or more times instead of counting unique references. It is more reliable to state that an interaction mechanism has been mentioned or not in an intervention than to determine if it has been mentioned three or four times in the same intervention. After the interview has been coded, to facilitate the analysis, the interaction mechanisms are grouped into the following categories.

- RF: Radio Frequency: any device that sends information wirelessly through electromagnetic signals. (keywords “rf”, “nfc”, “rfid”, “bluetooth”, “wifi”)
- Mobile: Usage of portable devices, be it through a smart phone or tablet app or a visitors guide device. (keywords “guide”, “tablet”, “app”)
- Video: Video or audio capturing devices. (keywords “video”, “ir-cameras”, “audio”)
- Self: Self reported mechanisms. Those where the visitor actively reports time spent or level of engagement by pressing a button or scanning a ticket or QR code next to the exhibitions. (keywords “happy-or-not”, “ticket”, “qr”)
- Others: Other interaction mechanisms mentioned by the students, combination of other different mechanism, Brain-Computer Interface, beacons or presence sensors. (keywords “combination”, “bci”, “beacon”, “sensor”)

## 4. Results

Two separate interviews were conducted with two groups each. One interview with the groups that had to use the QOC-FC framework and another interview with the students that had to use the PATH framework. The structure of the interview is given by the following topics in this order:

- Alternatives: What alternatives have the students found? Do the students consider there might be another alternative they have not thought about?
- Method: Which method have the students followed to elaborate the list of alternatives? How did they compare the alternatives? Do they consider that the framework was helpful? What were the pros and cons of the method or methods they used.
- Accuracy trade-off: Have the students considered any alternative that provides less amount of information than video recording? Do the students consider that another interaction mechanism different than video recording would be sufficient?
- Technical limitations dismissal: What alternatives could be considered if there was no technical limitation of what is possible with current technology?

**Figure 3:**
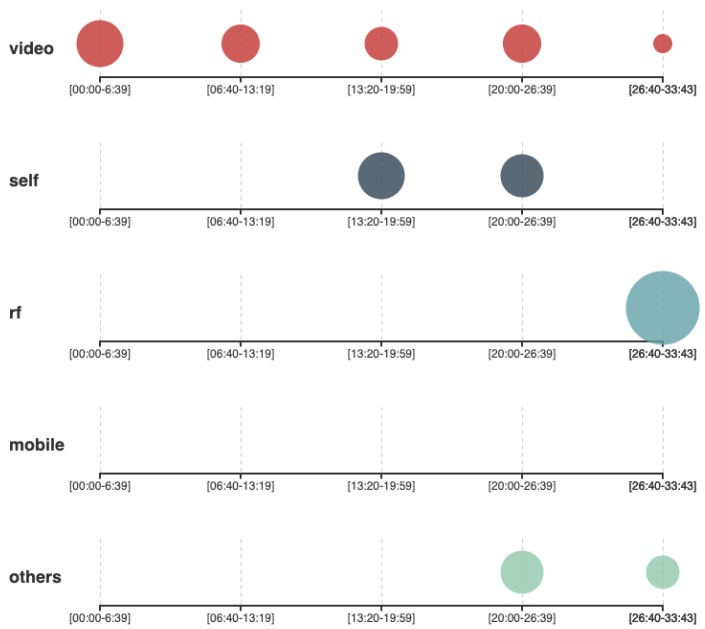
Frequency of mentions to different interaction mechanism by the QOC-FC groups.

**Figure 4:**
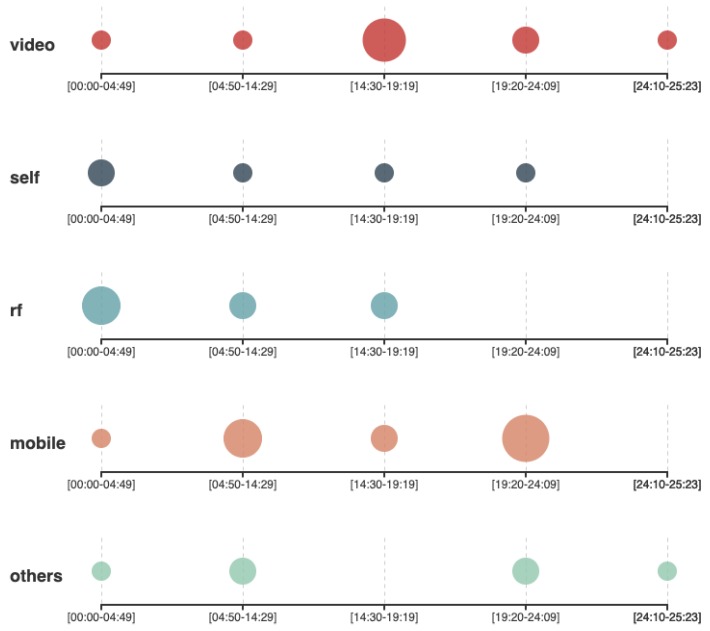
Frequency of mentions to different interaction mechanism by the PATH groups.

When requested to start discussing the alternatives they found to address the problem, the groups that used the QOC-FC framework (Group A and group B) provided a list of alternative methods, to guarantee that the video cameras in the museum will be noticed (following the guidelines of the QOC-FC framework to provide feedback about information capture), and methods to grant the museums’ visitors the possibility of disconnecting the cameras, in the case they had the preference of not being recorded (following the guidelines of the QOC-FC framework to provide the visitors with control over capture). Figure 3 shows that the mentions the students made to an interaction mechanism in the first half of the interview were mainly related to video capture. The group of students that used PATH (Figure 4) had a more distributed variety of mentions to different interaction mechanisms including the use of a feedback terminal, RFID, NFC, or the implementation of a mobile app to be used as a museums guide using a portable or stationary tablet.

At minute 15:45, the QOC-FC groups were asked if they had considered any alternative that did not involve video recording, even if that required to reduce the amount of information that could be captured. To this question, Group A answered that they did not really consider any other alternative to video recording, and Group B answered that they had considered only the feedback terminal (from the category of self reported interaction mechanisms). After that intervention from group B, the other interventions from the rest of the members started incorporating other types of interaction mechanism to the discussion, for example, the usage of the same entrance ticket to be scanned when using each exhibition. Asking the groups that used PATH if they had considered any interaction mechanism other than video recording was not applicable, as they already started discussing other interaction mechanisms from their first intervention. In the last part of the interview (Minute 25*’*26${}^{\prime \prime }$ for the QOC-FC interview and minute 22*’*36${}^{\prime \prime }$ of the PATH interview), the students are requested to be more open-minded and propose alternatives without considering any technical limitations. Both, QOC-FC and PATH groups propose an ideal technology capable of reading the mind [52] of the visitors. As there was not previous mention to any RF based interaction mechanism in the QOC-FC interview, groups A and B were explicitly asked by the interviewer if they were familiar with that technology. This question triggered a brainstorming session (Figure 3, minutes 26*’*40${}^{\prime \prime }$–33*’*43${}^{\prime \prime }$) where the students argued about the benefits and limitations of using Wifi or Bluetooth signals of the phone or a museum guide’s device to triangulate their position. The students then concluded that this might have been a good idea.

A more interpretative analysis of the interview was conducted in parallel with the quantitative analysis of the interaction mechanisms to address RQ2. Some terms and interventions, which were interesting or relevant to gain a better understanding of the students perspective about privacy, were annotated and labeled while coding the text of the interview. A total of 51 different tags were added to the text of the QOC-FC interview and 55 to the PATH interview. The more relevant tags were later grouped into the following categories: information, interview, PATH concept, QOC-FC concept, privacy concept, process, and privacy trade-off:

- Information: References to a type of information that the students considered relevant to capture from the museums visitors (age, gender, time spent, location, and engagement).
- PATH concept: A concept that is mentioned or explained by the PATH framework (intentionality, understandability, precision, etc.).
- QOC-FC concept: A concept that is mentioned or explained by the QOC-FC framework (capture, construction, control, feedback, and purpose).
- Privacy trade-off: A concept that the students identify as a trade-off between the privacy of the visitors and another factor (cost of implementation, architecture complexity, amount of information retrieved, and reward bias).
- Privacy concept: A concept related to privacy that is not mentioned in the two proposed frameworks and that is not considered a trade-off (consent and proportionality).

The privacy trade-offs identified by the students are of special interest. The cost of implementation is the trade-off that has been mentioned by the students more often, a total of 16 references for all the groups, even though it was not a requirement for the assignment. The students referred to this trade-off in terms of actual cost of the interaction mechanisms, needed equipment, or time required for the implementation. This concept is somehow related to the complexity of the architecture trade-off, as the more complex the system becomes, the more time would be necessary to implement it or to adapt it to the privacy requirements.

## 5. Discussion

The most immediate characteristic that can be observed from the scatter plots (Figure 3 and Figure 4) is that the distribution of points is considerably different for the treatment group and the control group. Even though there are mentions to video capture during the whole interview by all the groups, the PATH groups presented a more distributed variety of proposal of alternative implementations with different interaction mechanisms involved. The QOC-FC groups, however, spent more time describing specific aspects of video capture. This means that the QOC-FC group prematurely committed to using video capture based solutions as their technological approach. The explanation for this behavior is that the PATH groups were requested to conduct a simplified GOA as part of the design process, whereas the QOC-FC groups did not have that requirement. The recommendation from the PATH guidelines was to separate the problem description into three levels: **what**, **why**, and **how**. The problem description mentioned that Vitensenteret was interested in a system to monitor the behavior of the visitors (what), so that the quality of the exhibitions could be increased according to the interests of visitors (why) by using video recording techniques (how). Making the motivation explicit helped to interpret that the why was more important for Vitensenteret than the how, which could be considered only as a tentative suggestion. As Vitensenteret had not provided a solid reason for maintaining video capture as the only alternative, the students had the possibility to extend more on other less privacy intrusive technologies. For example, group C referred to the possibility of implementing a museum guide as a mobile app (Figure 4, mobile). As the app needed to be voluntarily downloaded, the visitor had a higher level of control over their privacy compared to the video capture scenario. Group C also argued that the application could also ask the visitors about their level of satisfaction for each exposition, allowing to estimate the engagement for the session. On the contrary, the QOC-FC groups addressed the problem by elaborating alternatives to provide the museums visitors with mechanisms for feedback (i.e., informing the visitors that they would be recorded) and control over their privacy (i.e., turning off the camera when a visitor requests not to be recorded), following the guidelines proposed by QOC-FC. Apart from the variety of interaction mechanisms proposed, the groups behaved in a similar way with other aspects of the experiment. For example, all the groups mentioned the same type of information they intended to obtain from the visitors (age, gender, level of engagement, location, and duration). They also identified similar trade-offs between the level of privacy they could guarantee to the visitors and other factors, like implementation cost, amount of information captured, and ease of use of the system. This was expected since none of the frameworks included any guideline or recommendation for these aspects of privacy.

To answer RQ1, as the PATH groups were able to hold a much broader set of design alternatives, avoiding the limitations imposed by the use of video capture as the only enabling technology, we consider that they responded more successfully to the challenge of facing an overspecification than the control groups. Our understanding is that applying a GOA at early stages of the system design is a crucial step in reducing the negative impact on privacy of overspecifications during the design of this type of applications. In the specific case of Internet of Things scenarios, restricting the how-level to the interaction mechanism simplifies the execution of the GOA, as the number of variables in the analysis is reduced to few concepts that are more understandable by the practitioners. For these reasons, we consider that PATH can be used effectively by people knowledgeable in general software development methods, but without expertise in privacy or privacy-enhancing technologies, to reduce the kind of overspecifications that might hurt user privacy.

With respect to RQ2 (What type of design decisions with potential negative impact on privacy should a privacy framework such as PATH help identify?), there are many different design factors that have an impact on users’ privacy. Both groups of students recognized that the privacy friendliness of the system is a trade-off between the available resources and what solution can be achieved. The cost of implementation is the trade-off that was mentioned most of the time during both interviews, even though the problem definition provided to the students did not include any mention or request to take into account the economic aspects in the design. This means that if students opted to implement any solution based on the low cost when there is no actual limit in the budget, the cost of implementation should be considered as an overspecification introduced by the same students during the design phase. For a privacy framework to be effective in preventing the incorporation of new overspecifications in the design, it should be applied in an iterative fashion and advising to take design decisions based on measurable factors (instead of wild guesses) and explicit requirements (instead of personal preferences).

Comparing our results to similar experiments performed by other authors is not trivial. Even though many privacy frameworks have been proposed in the literature, few empirical studies have been conducted to measure their effectiveness [53], mainly because it is a considerably costly process. Even from the few authors that describe empirical evaluations of their frameworks the focus of their research falls outside the areas of privacy-by-design, ubiquitous computing or overspecifications at the same time. One of the studies that share, at least, few aspects in common to our research is the experiment carried out by Jensen et al. [37], in which 32 students were recruited to compare the performance of two frameworks, Belloti and Sellen’s QOC-FC, and their proposed treatment STRAP. The Augur calendar system was used as the test application to be analyzed by the students because of its known set of privacy vulnerabilities. Similarly to PATH, the initial step proposed in STRAP was to conduct a goal-oriented analysis, however, it required to complete some additional steps, including the identification of actors, goals and system components for the elaboration of a goal-tree. Another significant difference with PATH was the type of questions that needed to be answered for each goal or sub-goal. The questions on STRAP were on the what-level (i.e., “What information is captured/accessed for this goal?”, “What knowledge is derived from this information?” and “What is done with the information afterward?”) and the who-level(i.e., “Who are the actors involved in the capture and access?”), while the questions on PATH where at the why-level (i.e., Why does the customer need to develop this application) and the how-level (i.e., How or how-else can this application be implemented). The type of results obtained in the experiment also difficult the comparison with the STRAP experiment. Jensen et al. focused on measuring completeness (i.e., finding all or as many as possible known privacy issues) while our focus was on the expansion of the design alternatives elaborated by the students (which is more open-ended).

On a more general level, our findings highlight the differences of the results obtained by each group depending on the method used to conduct the analysis. In other words, “a method or representation will enable the analyst to identify more threats in the areas where the method helps him to focus” [54]. This is in line with the experiment described by Iachello et al. [50] who stated that “different design methods for privacy highlight different sets of issues”. In their experiment, they evaluated four different frameworks: QOC-FC [35], Risk Analysis [55], QOC [36], and their own (proportionality method). None of the methods achieved a complete coverage of detected issues, instead, each method seemed to address different sections of the design space. For this reason, the authors considered all the methods as complementary (not one more efficient than others), proposing as a solution to elaborate a toolbox of privacy methods to select from, depending on, “the application domain, the deployment context, and the type of privacy and security issues involved”.

## 6. Threats to Validity

We have classified the possible threats to validity for this experiment, following the guidelines proposed by Wohlin et al. [56], in conclusion validity, internal validity, construct validity, and external validity. Each of them is presented in a separate subsection for clarity.

### 6.1. Conclusion Validity

In general terms, conclusion validity is threatened by the lack of statistical significance in experiments that count with a reduced sample, making it impossible to determine weather there are specific patterns associated to the data [56] (p. 185). As our intention is not to demonstrate any statistical hypothesis through a quantitative analysis, this type of threat is not so applicable to our case. However, the low number of participants (14 students arranged in four groups) has been an important limitation for us, and a larger sample would have been desirable in order to elaborate more robust claims. However, it is still possible to estimate the probability of obtaining the same results as in our experiment (The two PATH groups successfully challenging the overspecification and the two QOC-FC groups subjugated by the overspecification.) in a totally random scenario. We assume 50/50 probability ($p_{g}$ = 0.5) that a group can challenge the overspecification by providing at least one interaction mechanism as an alternative to video capture. Values much lower than 0.5 would mean that PATH is highly successful for dealing with overspecifications, whereas values much higher than 0.5 would mean that PATH is innocuous but QOC-FC considerably worsens the effect of the overspecification. The final probability of obtaining our results would be $p_{f}=p_{g}^{4}=0.0625$. In a worst-case scenario, considering a hypothetical diffusion of treatment, one of the PATH groups could have been influenced by the other treatment group (this issue does not apply to the QOC-FC groups, because, as observed, they did not propagate any alternative to video capture). The probability in this case would be $p_{d}=\left(1-{\left(1-p_{g}\right)}^{2}\right)\left(p_{g}^{2}\right)=0.1875$. On an individual basis, we can consider $p_{i}$ as the probability of a single student not being influenced by the overspecification and providing an alternative interaction mechanism. The value of $p_{i}$ should be large enough so that at least one student using PATH in a group of at least 3 students overcomes the challenge ($p_{path}$). This means that $p_{path}=1-{\left(1-p_{i}\right)}^{3}$ should be plausible. At the same time, $p_{i}$ should be small enough so that none of the QOC-FC students in a group could provide any alternative. Meaning that $p_{qoc}={\left(1-p_{i}\right)}^{3}$ should be plausible as well. A value of $p_{i}=0.206$ would make it equally possible for QOC-FC and PATH groups to behave as expected. The final probability of obtaining similar results considering the individual setup based on our groups configuration would be $p_{fi}=\left(1-{\left(1-p_{i}\right)}^{4}\right)\left(1-{\left(1-p_{i}\right)}^{3}\right){\left(1-p_{i}\right)}^{4}{\left(1-p_{i}\right)}^{3}$. That approximates to a 6% probability of obtaining the same results on a random scenario assuming that the methods QOC-FC and PATH did not have any effect on the participants. We believe that this probability is low and it is easier to conclude that $p_{i}$ is bigger for individuals using PATH than for individuals using QOC-FC ($p_{i-path}>p_{i-qoc}$)

We consider that the conclusion of this experiment has to be understood from the perspective of initial hypothesis validation that can lay the basis for an interesting future research.

### 6.2. Internal Validity

Any condition that might affect the dependent variable being observed (the variety of interaction mechanisms proposed by the students) without the researchers being aware supposes a threat to internal validity. Common threats of this type are selection bias, diffusion bias, maturation, timing effect, and experimenter bias.

- Selection bias: Assuming two types of subject: Type A, more prone to provide a wider variety of alternatives (i.e., because they have more extensive knowledge about technology, they are less biased against overspecifications or they have a more critical mindset), and type B, more prone to stick with the proposed alternative (i.e., because they have limited knowledge about technological alternatives, tend to be easily biased against overspecifications or simply are not motivated enough to make an effort to challenge the problem proposed). A selection bias would occur if any particular selection method promoted that individuals of type A were assigned the PATH method while individuals of type B were assigned the QOC-FC method. As the students were assigned one session or the other based on their availability, students and researchers did not know each other beforehand, and the students did not know any details about the experiment, it is not likely that this type of bias had an impact on the result. We could have applied a more thorough random selection method; however, that would have led us to mix both methods in the two sessions, endangering the result to be altered by a diffusion bias (treatment subjects observing or learning from control subjects or the other way around). Although the selection process was not biased in any way, it must be acknowledged that one group of students may have been better than the other one just by random chance. With the low number of participants, a small random unevenness of the two groups could have an effect. For instance, if there were two students who were a lot more competent than the rest (e.g., with a better understanding of privacy, stronger ability to challenge premature design assumptions), and both were to end up in the PATH section, perhaps each dominating a group in a positive direction, this—rather than a difference between the two approaches—could explain some of the differences in student results. At least, it can be said that from the observation of the work sessions of the experiment groups, all participants seemed to be eagerly contributing to the discussions, and none of the groups had individual students that seemed to be strongly dominating the discussions. Nevertheless, it cannot be completely excluded that the groups were different by chance, and this is a definite weakness that must be acknowledged from having a limited number of participants.
- Diffusion bias: As the two sessions of the evaluation took place in consecutive days, there was a chance of subjects who assisted in the first day leaking information to those who were going to assist the day after. To prevent this, the students were explicitly requested to avoid sharing information about the experiment with others. The second day, during the interview process, the students were asked a few key questions to ensure that they received no information from previous students.
- Maturation: As each participant was involved in the experiment once and no more than one sample was obtained from the same individual, there is no threat of maturation in the experiment.
- Timing effect: The two sessions took place on two consecutive working days (Thursday and Friday) from 10:00 to 14:00. No special dates or holidays took place during the week. We consider that the allocated time slots had a neglectable impact over the students’ cognitive performance.
- Experimenter bias: As one of the methods (PATH) was designed by the researchers, there was a high risk that an experimenter bias altered the results of the evaluation. We were especially aware of this and adopted a precautionary approach. An experimenter bias could be introduced in three different points during the evaluation, namely, task communication (i.e., by giving unconscious hints to the students about what type of response the researchers expected them to provide), interview (i.e., by leading the conversation towards a point in which the students reached the desired response), and data analysis (i.e., by focusing our attention on specific segments of the obtained data and ignoring others). It would have been ideal to request the intervention of an independent third party to conduct the experiment, however, the lack of resources was an impediment for this. Instead, we elaborated a thorough research protocol to avoid biases in the experiment. For the task communication, all the information was given to the students through a printed document (one page for the problem description and one page for the method to use, Figure 1 and Figure 2). The initial presentation consisted only of reading out loud the documents to the students. A special effort was put in not disclosing which method was our own. Since none of the methods are part of the study program the students were not aware of the authorship of both. The provided documents did not include any author information (i.e., names, affiliations, or dates). All documents were read in a uniform way so that the students did not suspect that one of the method had any preference over the other. Any questions from the students that could be solved by reading again parts of the document were answered this way. For questions about any other aspect that was not specified in the documents, the students were informed that they had freedom of choice on that matter. To avoid biases during the interview process, a semistructured approach was taken, following a script with the topics to prevent an induced response from the students. To prevent a biased analysis of the data, the whole interview was recorded and transcribed, using the method proposed by Bryman [51] (ch. 13) as described in Section 3.3.

### 6.3. Construct Validity

For our hypothesis, the ideal way of obtaining a faithful measure is to identify a real case scenario of the development of a ubiquitous computing application in which we knew beforehand that an overspecification is about to be introduced in the design, so that we could propose the development team to apply the PATH framework and observe if the resulting design is an improvement compared to how it would have been without applying the framework. The question would be if measuring the number of mentions of different interaction mechanisms in a system design session would be representative of how many design alternatives were evaluated as potential candidates. We estimate that the interaction mechanisms mentioned during the interview reflects the alternatives that were taken into consideration by the groups as viable to implement the system, independently of which solution was finally chosen. Two possible considerations are:

1. Would it be possible that a group provided a wide variety of alternatives to the design while still suffering the consequences of the overspecification? (false positive).
2. Would it be possible that a group provided only solutions based on the given alternative (video capture) even though they were not conditioned by the overspecification? (false negative)

Option (1) does not make sense since describing alternative methods other than video capture contradicts the (over) specification of using video capture. Option (2) could happen if, for example, all the members of a group acknowledged that there were other alternatives to video capture but still decided not to mention any of them. To prevent false negatives (option 2), the groups were explicitly asked if they had considered any other alternative that did not involve video capturing, to which they replied negatively.

### 6.4. External Validity

The main concern related to external validity is: to what extent can the results of the experiment be generalized? As Wohlin et al. [56] (p. 185) points out, the main threat for this type of validity is the use of students instead of professionals for the experiment. As it was discussed in Section 3, the use of students instead of experts is suitable for our research at the current state, since our intention is to validate an initial hypothesis at an early stage [42,44,45]. Wohlin also mentions that this threat is reduced if students are in their last year, near the completion of the studies and about to start a professional career in the industry (which is our case). It could be argued that either inexperienced professionals or students are likely to be more vulnerable to overspecifications than experienced professionals. As Shmueli et al. explain, overspecifications appear as the result of a cognitive bias, and, “findings about the influence of cognitive biases on behavior and decisions can be generalized from students to experts” [17] as, “Both students and managers are likely to have the similar cognitive limitations, so both groups are likely to exhibit bounded rationality in decision-making” [57]. This is also in line with our findings during the evaluation of the PATH framework with experts [19,20], expert system developers are also susceptible to get biased by overspecifications.

It could be argued, as well, whether the chosen scenario (the science museum) is representative of all the possible application domains where PATH could be used (interactive ubiquitous computing systems). As the time allocated for the experiment was limited, it was not possible to propose to the students a wide range of scenarios. However, the fact that the proposed problem is a real case scenario requested by the museum guarantees the applicability of the method in similar situations. Many commercial establishments might have the interest to survey the behavior and emotions of visitors/customers/clients in order to be able to analyze strong and weak points and make their services more attractive. Using video recording as a way to obtain behavioral information from customers seems to be a “hot topic” [58,59] and chances are that the PATH framework would be useful, at least, on those scenarios.

## 7. Conclusions and Future Work

In this work we conducted a controlled experiment with students in which they had to perform the same task using two different methods. One of the methods (the control) is a generic privacy analysis framework (QOC-FC) commonly used as a benchmark for similar experiments [53]. The other method (PATH) covers other privacy topics without overlapping with those covered by QOC-FC. The first step during the application of PATH is to conduct a simplified goal-oriented analysis, which is the main difference with respect to QOC-FC. In the problem description of the task assigned to the students, an overspecification (use of video cameras and computer vision) was added on purpose. The effect of this overspecification can be appreciated in the transcription of both interviews QOC-FC and PATH, since references to the video capture interaction mechanism are present throughout the whole interview (Figure 3 and Figure 4). However, the groups that used PATH also provided a wider range of design alternatives (some of them not as privacy intrusive as video capture), thus potentially reducing the impact on visitors’ privacy. The size of this experiment is still too low to draw a very robust conclusion. Nonetheless, the initial findings suggest that further research in this area might be promising.

There are some different possible paths for future work to continue in this line of research, for example:

- Conducting similar experiments with a larger number of participants and with a higher level of expertise.
- Comparing the PATH framework with other privacy methods. One possibility would be to compare PATH with STRAP, as both include GOA as part of the method, even though STRAP does not target overspecifications.
- Evaluating PATH over a long term experiment on a case study to better understand the iterative aspect of the framework. This way, it could be observed if the framework is still effective for managing overspecification while preventing that new types of overspecifications are introduced in the design.
- Investigate other types of overspecifications. So far, the PATH framework is designed to address overspecifications similar to the one proposed in the science museum scenario (overspecified interaction mechanisms). The cost of the implementation, for example, is another type of nonfunctional overspecification and it can be interesting since most of the groups interviewed were concerned about this matter.
- The PATH vocabulary can be extended with some of the attributes mentioned by the students: wearability, since wearable devices are more likely to expose personal information (i.e., location) than non-wearable devices and identifiability, as in the capability of an interaction mechanism of identifying the user.
- Propose other application scenarios with different types of interaction mechanism. The impact on users’ privacy might be different depending on the technology used to implement the system so it is interesting to find better ways to guide the design.

Further results in this area of research might be used to redesign the PATH framework to adapt it to the new findings and improve its applicability.

## Figures and Tables

**Table 1:** Students distribution.

Group	Number of Members	Method Used	Scenario
A	3	QOC-FC	Visitors Tracking
B	4	QOC-FC	Visitors Tracking
C	4	PATH	Visitors Tracking
D	3	PATH	Visitors Tracking

## Abbreviations

The following abbreviations are used in this manuscript.

BCI	Brain Computer Interface
NFC	Near Field Communication
GDPR	General Data Protection Regulation
IoT	Internet of Things
JAD	Joint Application Design
PATH	Privacy Aware Transmission Highway
QOC-FC	Question and Options Criteria-Feedback and Control
RAD	Rapid Application Development
RFID	Radio Frequency IDentification
STRAP	STRuctured Analysis of Privacy

## References

[1] Warren S.D., Brandeis L.D. (1890). The right to privacy. Harv. Law Rev..

[2] Westin A.F. (1967). Privacy and Freedom.

[3] Altman I. (1975). The Environment and Social Behavior: Privacy, Personal Space, Territory, and Crowding.

[4] Solove D.J. (2008). Understanding Privacy.

[5] Williams M., Nurse J.R., Creese S. Privacy is the boring bit: user perceptions and behaviour in the Internet-of-Things. Proceedings of the 2017 IEEE 15th Annual Conference on Privacy, Security and Trust (PST).

[6] Ziegeldorf J.H., Morchon O.G., Wehrle K. (2014). Privacy in the Internet of Things: threats and challenges. Secur. Commun. Netw..

[7] Pérez Fernández A. Towards the Tangible Hyperlink. Proceedings of the Seventh International Conference on Advances in Computer-Human Interactions, ACHI 2014.

[8] Hoepman J.H. Privacy design strategies. Proceedings of the IFIP International Information Security Conference.

[9] Spiekermann S., Cranor L.F. (2009). Engineering privacy. IEEE Trans. Softw. Eng..

[10] Langheinrich M. Privacy by design—Principles of privacy-aware ubiquitous systems. Proceedings of the Ubicomp 2001: Ubiquitous Computing.

[11] Yamin M., Alsaawy Y., Alkhodre A., Abi Sen A.A. (2019). An Innovative Method for Preserving Privacy in Internet of Things. Sensors.

[12] Yin X.C., Liu Z.G., Ndibanje B., Nkenyereye L., Riazul Islam S.M. (2019). An IoT-Based Anonymous Function for Security and Privacy in Healthcare Sensor Networks. Sensors.

[13] Cavoukian A. (2009). Privacy by Design: The 7 Foundational Principles. Information and Privacy Commissioner of Ontario, Canada. https://www.iab.org/wp-content/IAB-uploads/2011/03/fred_carter.pdf.

[14] Iachello G., Abowd G.D. Privacy and proportionality: adapting legal evaluation techniques to inform design in ubiquitous computing. Proceedings of the SIGCHI Conference on Human Factors in Computing Systems.

[15] Abdulghani H.A., Nijdam N.A., Collen A., Konstantas D. (2019). A Study on Security and Privacy Guidelines, Countermeasures, Threats: IoT Data at Rest Perspective. Symmetry.

[16] Abdul-Ghani H.A., Konstantas D. (2019). A Comprehensive Study of Security and Privacy Guidelines, Threats, and Countermeasures: An IoT Perspective. J. Sens. Actuator Netw..

[17] Shmueli O., Pliskin N., Fink L. (2015). Explaining over-requirement in software development projects: an experimental investigation of behavioral effects. Int. J. Proj. Manag..

[18] Shmueli O., Ronen B. (2017). Excessive software development: Practices and penalties. Int. J. Proj. Manag..

[19] Pérez Fernández A., Sindre G. (2018). The privacy aware transmission highway framework. Int. J. Inf. Priv. Secur. Integr..

[20] Perez Fernandez A., Sindre G. (2019). Software Assisted Privacy Impact Assessment in Interactive Ubiquitous Computing Systems.

[21] Bednar K., Spiekermann S., Langheinrich M. (2019). Engineering Privacy by Design: Are engineers ready to live up to the challenge?. Inf. Soc..

[22] Boehm B. (1996). Anchoring the software process. IEEE Softw..

[23] Jones C. (1996). Strategies for managing requirements creep. Computer.

[24] Yu E., Mylopoulos J. Why goal-oriented requirements engineering. Proceedings of the 4th International Workshop on Requirements Engineering: Foundations of Software Quality.

[25] Buschmann F. (2010). Learning from failure, part 2: featuritis, performitis, and other diseases. IEEE Softw..

[26] Coman A., Ronen B. (2010). Icarus’ predicament: managing the pathologies of overspecification and overdesign. Int. J. Proj. Manag..

[27] Simon H.A. (1957). Models of Man; Social and Rational.

[28] Shmueli O., Pliskin N., Fink L. (2016). Can the outside-view approach improve planning decisions in software development projects?. Inf. Syst. J..

[29] Hendrix T.D., Schneider M.P. (2002). NASA’s TReK project: A case study in using the spiral model of software development. Commun. ACM.

[30] Lee-Kelley L., Sankey T. (2008). Global virtual teams for value creation and project success: A case study. Int. J. Proj. Manag..

[31] Bjarnason E., Wnuk K., Regnell B. (2012). Are you biting off more than you can chew? A case study on causes and effects of overscoping in large-scale software engineering. Inf. Softw. Technol..

[32] Damian D., Chisan J. (2006). An empirical study of the complex relationships between requirements engineering processes and other processes that lead to payoffs in productivity, quality, and risk management. IEEE Trans. Softw. Eng..

[33] Choi K., Bae D.H. (2009). Dynamic project performance estimation by combining static estimation models with system dynamics. Inf. Softw. Technol..

[34] Gellman R. (2017). Fair Information Practices: A Basic History.

[35] Bellotti V., Sellen A. Design for privacy in ubiquitous computing environments. Proceedings of the Third European Conference on Computer-Supported Cooperative Work, ECSCW’93.

[36] MacLean A., Young R.M., Bellotti V.M., Moran T.P. (1991). Questions, options, and criteria: Elements of design space analysis. Hum.-Comput. Interact..

[37] Jensen C., Tullio J., Potts C., Mynatt E.D. (2005). STRAP: A Structured Analysis Framework for Privacy. https://smartech.gatech.edu/handle/1853/4450.

[38] Thomas K., Bandara A.K., Price B.A., Nuseibeh B. Distilling privacy requirements for mobile applications. Proceedings of the 36th International Conference on Software Engineering.

[39] ElShekeil S.A., Laoyookhong S. (2017). GDPR Privacy by Design. https://dsv.su.se/polopoly_fs/1.351720.1507815130!/menu/standard/file/Stipendie2017_ElShekeil-Laoyookhong.pdf.

[40] Gillham B. (2000). Developing a Questionnaire.

[41] Zainal Z. (2007). Case study as a research method. J. Kemanus..

[42] Sjøberg D.I., Hannay J.E., Hansen O., Kampenes V.B., Karahasanovic A., Liborg N.K., Rekdal A.C. (2005). A survey of controlled experiments in software engineering. IEEE Trans. Softw. Eng..

[43] Falessi D., Juristo N., Wohlin C., Turhan B., Münch J., Jedlitschka A., Oivo M. (2018). Empirical software engineering experts on the use of students and professionals in experiments. Empir. Softw. Eng..

[44] Höst M., Regnell B., Wohlin C. (2000). Using students as subjects—A comparative study of students and professionals in lead-time impact assessment. Empir. Softw. Eng..

[45] Svahnberg M., Aurum A., Wohlin C. Using students as subjects-an empirical evaluation. Proceedings of the Second ACM-IEEE International Symposium on Empirical Software Engineering and Measurement.

[46] Salman I., Misirli A.T., Juristo N. Are students representatives of professionals in software engineering experiments?. Proceedings of the 2015 IEEE/ACM 37th IEEE International Conference on Software Engineering.

[47] Diehl M., Stroebe W. (1987). Productivity loss in brainstorming groups: Toward the solution of a riddle. J. Pers. Soc. Psychol..

[48] Rietzschel E.F., Nijstad B.A., Stroebe W. (2006). Productivity is not enough: A comparison of interactive and nominal brainstorming groups on idea generation and selection. J. Exp. Soc. Psychol..

[49] Rodríguez D., Sicilia M.A., García E., Harrison R. (2012). Empirical findings on team size and productivity in software development. J. Syst. Softw..

[50] Iachello G., Abowd G.D. (2008). From privacy methods to a privacy toolbox: Evaluation shows that heuristics are complementary. ACM Trans. Comput.-Hum. Interact. (TOCHI).

[51] Bryman A. (2016). Social Research Methods.

[52] Wolpaw J.R., Birbaumer N., Heetderks W.J., McFarland D.J., Peckham P.H., Schalk G., Donchin E., Quatrano L.A., Robinson C.J., Vaughan T.M. (2000). Brain-computer interface technology: A review of the first international meeting. IEEE Trans. Rehabil. Eng..

[53] Huth D., Matthes F. “Appropriate Technical and Organizational Measures”: Identifying Privacy Engineering Approaches to Meet GDPR Requirements. https://aisel.aisnet.org/amcis2019/info_security_privacy/info_security_privacy/5/.

[54] Stålhane T., Sindre G. Safety hazard identification by misuse cases: Experimental comparison of text and diagrams. Proceedings of the International Conference on Model Driven Engineering Languages and Systems.

[55] Hong J.I., Ng J.D., Lederer S., Landay J.A. Privacy risk models for designing privacy-sensitive ubiquitous computing systems. Proceedings of the 5th Conference on Designing Interactive Systems: Processes, Practices, Methods, and Techniques.

[56] Wohlin C., Runeson P., Höst M., Ohlsson M.C., Regnell B., Wesslén A. (2012). Experimentation in Software Engineering.

[57] Seddon P.B., Scheepers R. (2012). Towards the improved treatment of generalization of knowledge claims in IS research: Drawing general conclusions from samples. Eur. J. Inf. Syst..

[58] Yamamoto J., Inoue K., Yoshioka M. Investigation of customer behavior analysis based on top-view depth camera. Proceedings of the 2017 IEEE Winter Applications of Computer Vision Workshops (WACVW).

[59] Paolanti M., Romeo L., Liciotti D., Pietrini R., Cenci A., Frontoni E., Zingaretti P. (2018). Person Re-Identification with RGB-D Camera in Top-View Configuration through Multiple Nearest Neighbor Classifiers and Neighborhood Component Features Selection. Sensors.

